# Bullying of Medical Students in Pakistan: A Cross-Sectional Questionnaire Survey

**DOI:** 10.1371/journal.pone.0003889

**Published:** 2008-12-08

**Authors:** Syed Ahmer, Abdul Wahab Yousafzai, Naila Bhutto, Sumira Alam, Amanullah Khan Sarangzai, Arshad Iqbal

**Affiliations:** 1 Department of Psychiatry, Aga Khan University, Karachi, Pakistan; 2 Department of Psychiatry, Services Institute of Health Sciences, Lahore, Pakistan; 3 Department of Medicine, Aga Khan University, Karachi, Pakistan; 4 Aga Khan University Medical College, Karachi, Pakistan; Aga Khan University, Pakistan

## Abstract

**Background:**

Several studies from other countries have shown that bullying, harassment, abuse or belittlement are a regular phenomenon faced not only by medical students, but also junior doctors, doctors undertaking research and other healthcare professionals. While research has been carried out on bullying experienced by psychiatrists and psychiatry trainees in Pakistan no such research has been conducted on medical students in this country.

**Methodology/Principal Findings:**

We conducted a cross-sectional questionnaire survey on final year medical students in six medical colleges of Pakistan. The response rate was 63%. Fifty-two percent of respondents reported that they had faced bullying or harassment during their medical education, about 28% of them experiencing it once a month or even more frequently. The overwhelming form of bullying had been verbal abuse (57%), while consultants were the most frequent (46%) perpetrators. Students who were slightly older, males, those who reported that their medical college did not have a policy on bullying or harassment, and those who felt that adequate support was not in place at their medical college for bullied individuals, were significantly more likely to have experienced bullying.

**Conclusion:**

Bullying or harassment is faced by quite a large proportion of medical students in Pakistan. The most frequent perpetrators of this bullying are consultants. Adoption of a policy against bullying and harassment by medical colleges, and providing avenues of support for students who have been bullied may help reduce this phenomenon, as the presence of these two was associated with decreased likelihood of students reporting having being bullied.

## Introduction

While bullying has been variously defined by different organizations and individuals, one useful definition is that it is a “persistent behaviour against an individual that is intimidating, degrading, offensive or malicious and undermines the confidence and self-esteem of the recipient”.[1]

Medicine is a very hierarchical profession and medical students are at the bottom of that hierarchy. A number of studies have shown that bullying, abuse, harassment, mistreatment or belittlement affect not only medical students [2]–[7], but also doctors in training [8]–[11], doctors undertaking research [12] and other healthcare professionals. [13] The estimates of experience of mistreatment, bullying or harassment experienced by medical students ranged from 42% [2] to 91% [4] depending on the definition of bullying used.

It has been suggested that more often accusations of bullying are linked to situations which are upsetting but an inevitable part of training, for example, trainers telling trainees that they are not performing at the expected level.[14] However, research has clearly shown that perceived mistreatment is viewed by medical students as a major source of stress[10]. More than a third of medical students in a study reported that they had considered dropping out and about a fourth of them reported that they would have chosen a different profession had they known about the extend of bullying in medical schools.[15] The experience of bullying and harassment has also been shown to be associated with long-term severe adverse mental health consequences, including depression and suicide attempts in doctors.[15]–[18]

There has been some research assessing extent of bullying or harassment faced by psychiatry trainees [11] and consultant psychiatrists [19] in Pakistan. However, to our knowledge no research has been conducted as yet studying the prevalence of bullying experienced by medical students in Pakistan.

## Methods

### Study design

This was a cross-sectional questionnaire survey.

### Sampling Method and Ethical Concerns

This study is part of a larger study carried out to assess multiple aspects of medical students' experiences of medical college life. Our sample consisted of final year students enrolled in six medical colleges in Pakistan. The names of these medical colleges are being withheld to preserve the anonymity of the participants.

A formal ethical review of this study was not sought as this study was non-experimental in nature and was a voluntary, anonymous survey of consenting adults. However, all possible ethical concerns were discussed with the faculty in the department of psychiatry, Aga Khan University, Karachi. Written Informed consent was obtained from all respondents. No participant-identifiable data was recorded to maintain confidentiality. All ethical concerns of the Helsinki Declaration were followed.

### Data Collection

There were two medical colleges from North West Frontier Province (NWFP), two from the province of Sind, one from the Punjab, and one from Balochistan. In each medical college we identified a key person, a senior postgraduate trainee or a consultant, who contacted the students and collected the data. The forms were given out and collected at the end of lectures.

We used the same data collection tool as used by the British Medical Association in its Medical Students Welfare Survey.[3] The data was collected on two forms. The first collected data on basic sociodemographic details and asked whether the respondent had been a victim of bullying or harassment whilst at medical school. Those respondents who replied in affirmative to the above question were asked which forms of bullying behaviours they had experienced, who did they face the bullying from, whether the bullying had been resolved or was on-going, whether the medical college had a policy on bullying and harassment and whether there was adequate support for bullied individuals.

The other questionnaire asked the students to what extent their health and wellbeing had been affected by work, study or stress and what were the sources of this stress. They were further asked if they were aware of use of alcohol, drugs or smoking to cope with stress, if they were aware of depression or use of antidepressants in medical students, and if they had received adequate teaching on substance misuse. This part of the study would be reported in a separate paper.

### Data Analysis

The data were analysed using Statistical Package for Social Sciences (SPSS) version 16.0. We used chi-square to analyse associations between categorical variables and t-test for continuous variables. We used logistic regression to find out which factor(s) best predicted the likelihood of having experienced bullying or harassment.

## Results

Out of a total number of 540 medical students enrolled in the final year of the six medical colleges at the time of the study 342 agreed to participate in the survey giving rise to a response rate of 64.5%. The mean age of respondents was 23.73 years (SD 2.45 years), 52.5% were male and 90% were single. Eighty-two percent of the respondents were enrolled in government-run medical colleges. Fifty-two percent of the respondents said they were from an urban background. Ninety-one percent of the respondents were Muslims.

About 48% of the respondents said that they had never been bullied in medical college. Among the rest of the 52%, 25% reported being bullied less than once a month, 15.9% once a month, 11% once a week, and 0.6% daily.

Among those who had faced bullying or harassment 56.9% reported that they had faced verbal abuse, 25.7% reported other behavioural gestures they perceived as representing bullying or harassment, 15.6% felt they had been deliberately ignored, 10.9% felt they had been excluded, 5% reported physical abuse and 2.5% written abuse. (The percentage adds upto more than 100 as some respondents reported facing more than one type of bullying behaviour)

About half (46%) of those who had experienced bullying or harassment said they had faced this behaviour from a consultant. Twenty five percent had faced this behaviour from postgraduate trainees/resident medical officers, about 18% each from nurses and peers, and 8.5% from patients. When asked if the bullying/harassment or was on-going 61% of the respondents said it was on-going. When asked if their medical college had a policy on bullying and harassment 88% replied in negative. Fifty-five percent of respondents felt that adequate support for individuals who had been bullied/harassed was not available at their medical college.

For purposes of analysis we collapsed the categories of having experienced bullying or harassment never, less than once a month, once a month, once a week and daily into a binary yes/no outcomes, the latter incorporating all other responses except never. This was used as the primary outcome variable.

The association between demographic variables and having faced bullying or harassment is shown in **Table 1**. Males, those who reported that their medical college did not have a policy on bullying/harassment, and those who felt that adequate support was not available for individuals who had been bullied/harassed, were significantly more likely to have experienced bullying or harassment. Respondents who had experienced bullying or harassment were slightly older than those who hadn't (mean 24.04 yrs vs 23.41 yrs respectively) and the difference was statistically significant (t = −2.295, P = 0.02).

**Table 1:** Demographic characteristics of participants experiencing bullying in medical college and its association with the likelihood of being bullied or harassed

Variable	Number (% of total sample)	Bullied (% within variable)	P-value
**Gender** (*n* = 303)			
Male	156 (52.3)	94 (60.3)	0.02*
Female	142 (47.7)	66 (46.5)	
**Marital status** (*n* = 265)			
Married	23 (8.7)	11 (47.8)	
Single	239 (90.2)	136 (56.9)	0.11
Divorced	3 (1.1)	0 (0)	
**Religion** (*n* = 312)			
Muslims	285 (91.3)	150 (52.6)	0.84
Non-Muslims	27 (8.7)	15 (55.6)	
**Type of Medical College** (*n* = 297)			
Public sector	244 (82.2)	131 (53.7)	0.45
Private	53 (17.8)	25 (47.2)	
**Background** (*n* = 278)			
Urban	147 (52.9)	82 (55.8)	0.81
Rural	131 (47.1)	71 (54.2	
**Policy on bullying** (*n* = 266)			
Yes	32 (12)	11 (34.4)	0.01*
No	234 (88)	141 (60.3)	
**Adequate support available** (*n* = 318)			
Yes	56 (17.6)	17 (30.4)	
Sometimes	87 (27.4)	39 (44.8)	<0.0001*
No	175 (55.0)	113 (64.6)	

* Statistically significant at the level of P<0.05

*n* is different for different variables as not all the students had answered all the questions

We performed subgroup analysis on whether there was a difference between genders on who they had faced the bullying from. Consultants were significantly more likely to bully male students than female students (50% vs 25.2%) while female students were more likely than male students to be bullied by nurses (13.6% vs 8.9%), patients (5.8% vs 1.6%), peers (17.5% vs 11.3%) and residents (17.5% vs 8.9%). Overall, the difference was statistically significant (p = 0.11)

We performed simple multiple logistic regression to find out which variable was the strongest predictor of a student experiencing bullying or harassment, using bullied/not bullied as the dependent variable. We entered all the variables that had yielded a p-value of less than 0.05 on t-test and chi square tests into the regression equation. These variables were age, gender, whether the medical school had a policy on bullying and harassment, and whether participants believed there was adequate support available for students who had been bullied or harassed. All the variables were entered into the regression equation at the same time.

The results of the multiple logistic regression analysis are shown in **Table 2**. Believing that adequate support was not available for bullied students, and belonging to male gender were the strongest predictors of having experienced bullying or harassment, respectively. Age, and the medical college having a policy against bullying and harassment, which were statistically significant on t-test and chi-squared tests respectively, were not significant on logistic regression.

**Table 2:** Factors associated with an increased likelihood of having experienced bullying or harassment: Simple Multiple Logistic Regression Analysis

Variables	Odds Ratios* (95% C. I.)	*P* Value
Age	1.09 (0.96–1.23)	0.20
Gender	0.52 (0.29–0.93)	0.03*
Policy on bullying	1.36 (0.49–3.76)	0.56
Adequate support for bullied individuals	1.90 (1.20–3.08)	0.006*

* Statistically significant at the level of P<0.05

## Discussion

In this first survey of bullying or harassment faced by medical students in Pakistan about half (52%) of the final year medical students surveyed reported that they had been bullied or harassed whilst at medical school. The most common form of bullying was verbal abuse (57%). The students reported that consultants were the most frequent (46% of cases) perpetrators of bullying. Older age, male gender, absence of an anti-bullying policy at the medical college, and perception of lack of support for bullied individuals, were all statistically significantly associated with increased likelihood of being bullied.

In a study from United States 42% of medical students reported harassment and 84% belittlement during their stay in medical school.[2] Two studies from Finland reported that from half to three–quarters of medical students surveyed reported experiencing some kind of mistreatment during their medical education.[5], [7] However, in a recent British Survey only 17% of medical students reported that they had been a victim of bullying or harassment during their stay at medical school.[3] The 52% prevalence of bullying experienced by students in our study is very close to the American and Finnish studies but much higher than the British study.

In our study overall male students were significantly more likely than female students to have experienced bullying. However, on subgroup analysis males were more likely than females to be bullied by consultants only, while females were more likely to be bullied by nurses, patients, peers and residents. Because consultants were responsible for about half of the bullying experienced by respondents this resulted in more males reporting bullying overall.

In most previous studies on this topic females were more likely to be bullied than males [5], [6], [16], [20]–[22] except for only two previous studies.[2], [7] In the study by Frank et al [2] gender was marginally significant only for harassment by preclinical professors (males vs females, 11% vs 7%) but not for harassment by all other groups. In Uhari's study [7] male students reported significantly more harassment by classmates, preclinical teachers, clinical teachers, clinicians and patients. Studies that have examined general abuse or harassment find a less striking difference than studies looking specifically at sexual harassment which is generally found to be suffered more commonly by female students.[2] In our study we had not asked specifically about sexual harassment or gender-based mistreatment so it is difficult to comment whether that would have influenced the results.

Students reporting that they had experienced bullying or harassment were slightly older that those who had not (mean 24.04 yrs vs 23.41 yrs respectively). However, the difference in their ages was so small that it was difficult to draw any conclusions from it.

The finding that the perception of adequate support not being in place for bullied individuals at their medical college was the strongest predictor of someone having experienced bullying is an important one. Does this mean that individuals who feel unsupported are more likely to be bullied or does it reflect on the overall culture of that medical college? This needs to be explored further.

The strengths of our study include its reasonably large sample size, recruiting students from all four provinces of Pakistan, and use of a data collection instrument that had already been used by the British Medical Association in their survey of medical students in the UK. We did not collect data on those students who chose not to participate in this study. This raises the possibility of those who had experienced bullying being more likely to participate in the survey and inflating the prevalence. However, considering that 48% of respondents reported that they had never experienced bullying or harassment, we think that this may not have greatly influenced the results. In Pakistan, many medical colleges have opened in the private sector in the past decade. We are not aware what proportion of medical students in Pakistan are studying in private medical colleges but in our study only about 18% of respondents were enrolled in private medical colleges. This may limit generalisation of our study's results to all medical students in Pakistan.

Consultants were the single largest group (46%) of perpetrators of bullying in our study. This finding is almost a universal one.[2]–[5], [11] Medical students learn not only medicine but also behaviour patterns of their seniors and mentors, the so-called ‘hidden curriculum of undergraduate medical education’. [23] Unless the medical profession is made more aware of what bullying is and how it affects those who experience it, it is likely that the cycle of bullying will continue. Two factors that participants in our study clearly identified as being protective against bullying were a medical college having a policy against bullying and harassment, and perception of availability of support for affected individuals. These may be a good starting point if we wish to curb bullying in our medical colleges.
